# Evolution and Population Structures of Prevalent Methicillin-Resistant *Staphylococcus aureus* in Taiwan

**DOI:** 10.3389/fmicb.2021.725340

**Published:** 2021-09-14

**Authors:** Chih-Jung Chen, Tsai-Ling Yang Lauderdale, Yhu-Chering Huang

**Affiliations:** ^1^Division of Pediatric Infectious Diseases, Department of Pediatrics, Chang Gung Memorial Hospital, Taoyuan, Taiwan; ^2^Molecular Infectious Diseases Research Center, Chang Gung Memorial Hospital, Taoyuan, Taiwan; ^3^School of Medicine, College of Medicine, Chang Gung University, Taoyuan, Taiwan; ^4^National Institute of Infectious Diseases and Vaccinology, National Health Research Institutes, Zhunan, Taiwan

**Keywords:** methicillin-resistant *Staphylococcus aureus*, whole-genome sequencing, evolution, time-scaled phylogeny, Taiwan

## Abstract

Global methicillin-resistant *Staphylococcus aureus* (MRSA) strains were dominated by few genetic lineages, suggesting their inherent advantage of competitive fitness. The information of genome evolution and population structures of prevalent MRSA strains can help gain a better understanding of the success of the pandemic clones. Whole-genome sequencing was performed in 340 MRSA isolates belonging to three prevalent lineages, including ST59 (129 isolates), ST239/241 (140 isolates), and ST5 (71 isolates), collected from 1996 to 2016 in Taiwan. The time-scaled phylogeny and evolutionary pathways were estimated by Bayesian analysis using Markov chain Monte Carlo. The toxome, resistome, and plasmids were characterized by screening the raw reads with a public database. ST59, ST239/241, and ST5 MRSA were estimated to emerge in 1974, 1979, and 1995, respectively, in Taiwan. ST59 evolved through two major pathways, generating two subclones in 1980 and 1984. Both ST59 subclones remained prevalent in the healthcare and community environments in late 2010s. ST239/241 diverged into three subclones, respectively, in 1989, 1993, and 1995. The 1995-emerging ST239 subclone predominated after 2000 by replacing two previous early subclones. ST5 could be subdivided into two clades within 3 years of introduction, but no substantial difference of genomic profiles was identified in the strains of distinct clades. Each of the three pandemic MRSA lineages harbored its own specific toxome, resistome, and plasmids. The frequently identified genetic diversities between the subclones of the same lineage were genes mediating immune evasion, leukocidins, enterotoxins, and resistance to aminoglycosides. In conclusion, MRSA ST59 and ST239/241 emerged in the 1970s and evolved drastically during 1980 and 1995, resulting in three successful subclones prevailing in Taiwan. ST5 was introduced late in 1995 without a significant genetic drift during 20 years of evolution.

## Introduction

*Staphylococcus aureus* exhibiting resistance to semi-synthetic penicillins (i.e., methicillin and oxacillin) was first reported in 1960 (Knox, 1960). The methicillin-resistant *S. aureus* (MRSA) remained uncommon (<5%) for two decades after its first documentation and was increasingly reported in healthcare facilities worldwide after 1980. In the United States, the rates of MRSA in large urban centers were between 5 and 10% in the mid-1980s and increased to a level of 40–50% by the end of the 1990s (Chambers, 2001). It was intriguing to learn that more than 70% of the MRSA isolates spreading in hospitals worldwide belonged to several successful lineages (Oliveira et al., 2001, 2002; Enright et al., 2002). A majority of the international MRSA isolates were believed to have evolved from only two ancestral genetic backgrounds, respectively, belonging to clonal complex (CC) 8 and CC5 (Oliveira et al., 2001). The Brazilian clone and Hungarian clone were common nosocomial MRSA clones of CC8, whereas the New York/Japan clone was the prevalent clone of CC5. The Brazilian clone was originally disseminated in Brazilian hospitals and later transmitted to several countries in Europe [also known as ST239/241 as defined by multi-locus sequence typing (MLST) and carrying a type IIIA staphylococcal chromosomal cassette *mec* (SCC*mec*)]. The Hungarian clone was widely spreading in Hungarian hospitals since 1993 and was reported to be the major MRSA clone in Taiwan and China in the l990s (ST239/241-SCC*mec*III). The New York/Japan clone was prevalent in New York, Connecticut, New Jersey, and Pennsylvania in the United States and was reported in a Tokyo hospital (ST5-SCC*mec*II) (Oliveira et al., 2002; de Sousa and Lencastre, 2004). The other known lineages of the healthcare-associated (HA) MRSA included CC30 (ST30, ST36, etc.), CC45 (ST45, ST46, etc.), and CC22 (ST22, etc.) (Enright et al., 2002; Gomes et al., 2006).

The novel strains of community-associated (CA) MRSA emerged in the late 1990s and was increasingly reported in many developed countries worldwide in the following decade (David and Daum, 2010; DeLeo et al., 2010). The incidences of MRSA in CA *S. aureus* infection reached greater than 50% in many regions of the world in the late 2000s (Kaplan et al., 2005; Huang et al., 2008). The CA-MRSA strains were characterized with a limited spectrum of resistance to non-beta-lactams, carriage of relatively smaller SCC*mec* elements (i.e., SCC*mec* types IV, V, VII, and VIII) and Panton-Valentine leukocidin (PVL) genes, and predominately associated with skin and soft tissue infections. The well-known epidemic CA-MRSA clones included ST8 (USA300) and ST1 (USA400) in North America, ST30 in South America, Southeastern Asia, and Oceania, ST80 in Europe, and ST59 in Asian countries including Taiwan (Kennedy et al., 2008; Li et al., 2009; Chuang and Huang, 2013; Chen and Huang, 2014).

Similar to the epidemiology in western countries, MRSA was rarely identified in Taiwan until the 1980s. The rate of HA-MRSA increased significantly in the 1980s and reached a high plateau level at 60–70% in the late 1990s and early 2000s (Chang et al., 1997; Chen et al., 1999; Hsueh et al., 2002). It has been shown that the Hungarian clone (ST239/241-SCC*mec*III) was the most prevalent MRSA clone accounting for more than 70% of HA-MRSA blood isolates collected island-wide in 2000–2001 (Huang et al., 2004). None of the other minor clones accounted for more than 10% of the sampled isolates during that period of time. However, the ST239/241 clone gradually lost its predominance in the following 10 years. In another study characterizing 577 island-wide MRSA blood isolates in 2010, the proportion of ST239/ST241 strains decreased from more than 70% to approximately 40% (Chen et al., 2014). The waning dominance of the Hungarian clone was accompanied by the emergence of another pandemic HA-MRSA clone, the New York/Japan clone of ST5-SCC*mec*II, and a significant increase of the epidemic CA-MRSA clones ST59-SCC*mec*IV and ST59-SCC*mec*V_T_ (Chen et al., 2014).

To better understand the population genomics, emergence, and evolution of the prevalent MRSA strains in Taiwan, a total of 340 MRSA isolates collected island-wide during 1996 and 2016 were characterized by whole-genome sequencing (WGS). The timeline of the molecular evolution for each lineage was estimated with Bayesian modeling and Markov chain Monte Carlo algorithm.

## Materials and Methods

### The Strains

In this study, we identified MRSA ST239/241, ST59, and ST5 isolates retrospectively from two collections of MRSA isolates, which included a total of 6,952 clinical MRSA isolates. The MRSA strains were routinely determined for pulsotypes or *spa* types, and some strains were also determined for MLST. The distribution of isolates with pulsotypes/*spa* types inferring to or that had been determined as one of the three major MLSTs (ST239/241, ST59, and ST5) in different years of collection are displayed in Tables 1, 2. The number of isolates selected for WGS in this study is also shown in detail. Totally, 71 ST5 isolates, 129 ST59 isolates, and 140 ST239 isolates, respectively, were selected for WGS. The first collection included MRSA isolates from the Taiwan Surveillance of Antimicrobial Resistance (TSAR) surveys. TSAR is a surveillance project, which was conducted by the investigators in the National Health Research Institute of Taiwan (Wang et al., 2016). TSAR collected the clinical isolates of various medically important bacterial species for the determination of antimicrobial resistance from up to 44 major hospitals island-wide in Taiwan biennially since 1998. A total of 3,766 clinical MRSA isolates were collected from the first nine surveys between 1998 and 2014, ranging from 243 isolates in the 2000 survey to 476 isolates in the 2012 survey (Table 1). A total of 32 ST5 isolates, 38 ST59 isolates, and 36 ST239 isolates, respectively, were selected for WGS, with three to five isolates from each survey if identified.

**TABLE 1:** Distribution of methicillin-resistant *Staphylococcus aureus* (MRSA) isolate total numbers, numbers of ST5, ST59, and ST239/241 isolates in different years of surveillance, and numbers of isolates for whole-genome sequencing analysis selected from the Taiwan Surveillance of Antimicrobial Resistance program collection.

**Year**	**Number of MRSA collected and characterized**	**Number of sequenced isolates/number of total isolates**		
		**ST5**	**ST59**	**ST239/241**
1998	373	0/0	5/50	4/161
2000	243	3/4	4/62	4/160
2002	495	4/13	4/200	4/253
2004	466	4/55	5/188	4/202
2006	407	3/55	5/158	4/169
2008	475	5/68	5/213	4/150
2010	390	5/40	4/162	4/108
2012	476	4/50	2/167	4/153
2014	441	4/23	4/166	4/113
Total	3,766	32/308	38/1,376	36/1,469

**TABLE 2:** Distribution of clinical methicillin-resistant *Staphylococcus aureus* isolate total numbers, numbers of ST5, ST59, andST239/241 isolates in different years of collection, and numbers of isolates selected for whole-genome sequencing analysis in the Chang Gung Memorial Hospital-based collection.

**Period/population**	**Total**	**Number of sequenced isolates/number of total isolates**		
		**ST5**	**ST59**	**ST239/241**
Year of the first isolate		1997	1998	1996
1995–2001	814	8/11	26/131	32/629
2002–2007	711	9/22	22/322	26/316
2008–2009	181	3/21	8/88	8/54
2010–2012	1,177	18/153	20/551	21/290
2013–2016	260	1/5	15/171	17/47
Total	3,143	39/212	91/1263	104/1,336
Pediatric	1,105	4/6	46/693	46/289
Adults	630	8/90	7/230	13/225
Unclassified	1,408	27/116	38/340	45/822

The second collection included MRSA isolates from a Chang Gung Memorial Hospital (CGMH)-based laboratory. For the surveillance of the molecular epidemiology of MRSA in Taiwan, we collected and molecularly characterized (by pulsed-field gel electrophoresis with or without MLST method) a total of 3,186 clinical MRSA isolates island-wide in Taiwan between 1995 and 2016 in the laboratory in CGMH (Table 2). In addition to CGMH, the isolates were also collected from at least 10 hospitals island-wide in Taiwan. A total of 1,135 isolates were identified from pediatric patients, 60 isolates from adult patients, and 1,421 isolates from all age groups for the surveillance of molecular epidemiology of MRSA in Taiwan (we estimated that more than 90% of the isolates were from adult patients). The isolates were identified from all body sites, and most isolates were estimated to be healthcare-associated. A total of 41 ST5 isolates, 91 ST59 isolates, and 104 ST239 isolates, respectively, were selected for WGS from the CGMH collection (Table 2).

### Whole-Genome Sequencing

The chromosomal DNA of the bacteria was extracted using QIAamp DNA Mini Kit (Qiagen, CA, United States). The WGS of the strains was performed with the Illumina MiSeq sequencer (Illumina, San Diego, CA, United States). The raw data of each strain were processed with adapter trimming and exclusion of reads in which greater than 45% of the bases were of quality score <20 (<Q20). A range of 199–318 million bases was generated for each strain, with 71.1- to 113.6-fold base coverage across the genome. This Whole Genome Shotgun project has been deposited at GenBank under accession number PRJNA695114. The analysis strategy was carried out as detailed in the following discussion.

### *De novo* Assembly of WGS

The *de novo* assembly of each of the strains was performed using SPAdes 3.11.1 (Bankevich et al., 2012). The default k-mer lengths of 21, 33, 55, 77, 99, and 127 were set, and *careful* option was used during the assembly procedure. Only the scaffold outputs with length greater than 200 bp were used in the following analysis.

### Multi-Alignment of Core Genomes and Time-Scaled Phylogeny

The multi-alignment of the core genomes of the MRSA strains was conducted separately for each of the three lineages (ST239/ST241, ST59, and ST5) with the parsnp program (Treangen et al., 2014). The reference strains used during the procedure were N315 (NC_002745.2) for ST5, TW20 (NC_017331.1) for ST239/ST241, and SA957 (NC_022442.1) for ST59. The core-genome covered an average of 85.2, 85.9, and 78.1% for the whole-genome lengths of the ST239/241, ST59, and ST5 strains, respectively. The multi-alignment files generated by the parsnp script were subsequently used for the construction of time-scaled phylogeny by the Bayesian analysis of the molecular sequences using Markov chain Monte Carlo. The BEAST program (v 1.10.4) was used to estimate the clock rates of evolution and the time to most recent common ancestor (MRCA) (Drummond and Rambaut, 2007). A HKY model with estimated base frequencies, uncorrelated relaxed clock type with lognormal distribution, and constant size of tree prior was used. Three independent chains were run for 100 million generations and sampling for every 1,000 generations. The effect sample size values were checked by tracer (v1.7.1), and all of them were greater than 200. A burn-in of 10 million states was removed from each of the three independent runs. The logcombiner program (v 1.10.4) from the BEAST package was subsequently used to combine the results from those runs. The final tree was output and annotated with the Figtree program.^1^

### Screening of Interested Genes

The carriage of plasmids, resistance, and virulence genes among the MRSA strains was detected by srst2 procedure using the PlasmidFinder (https://github.com/katholt/srst2/blob/master/data/PlasmidFinder.fasta, assessed on December 4, 2017), ARG-ANNOT (version 2, https://github.com/katholt/srst2/tree/master/data/ARGannot_r2.fasta, assessed on December 4, 2017), and virulence factor of pathogenic bacteria database Staphylococcus_VF (Virulence factors of Pathogenic Bacteria website, http://www.mgc.ac.cn/VFs/main.htm, Staphylococcus_VF_clustered.fasta, assessed on August 8, 2017), respectively (Inouye et al., 2014).

## Results

### Time-Scaled Phylogeny

#### ST239/241

**FIGURE 1:**
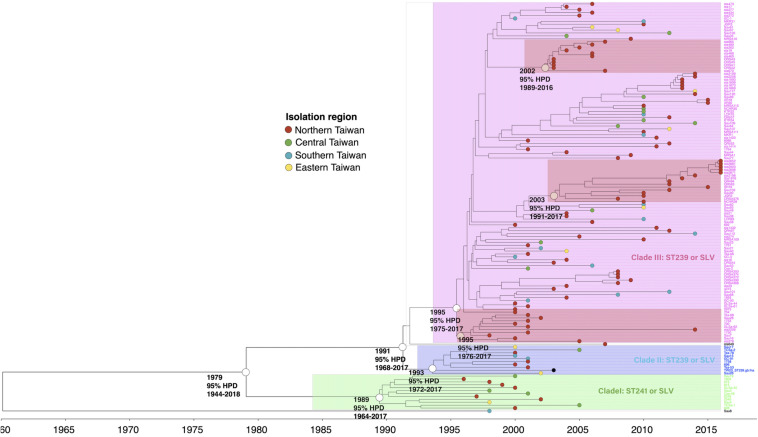
Time-scaled phylogeny of 140 methicillin-resistant *Staphylococcus aureus* isolates of the ST239/241 lineage collected in Taiwan during 1996 and 2016. Three major clades were marked with different colors. The hollow circles indicate the most recent common ancestors of the isolates of the indicated clade and its estimated year of appearance. The isolates of three subclades that emerged in 1995, 2002, and 2003 (marked as dark red) were identified in northern Taiwan, suggesting a local transmission of the strains. HPD, highest posterior density; SLV, single-locus variants.

The phylogenetic analysis of WGS indicated that the ST239/241 MRSA isolates could be divided into three major clades, respectively, of I (12 isolates, 8.6%), II (10 isolates, 7.1%), and III (116 isolates, 82.9%, Figure 1). All isolates except for one strain (Sau8) shared an MRCA in the year of 1979 [95% highest posterior density (HPD) 1944–2018]. The clade I isolates were estimated to emerge in 1989 (95% HPD 1964–2017) and were all belonging to ST241 or its single locus variants (SLVs). All 12 isolates of clade I were isolated before or in 2005. Clade II and clade III isolates all belonged to ST239 or its SLVs and with the MRCA appearing in the years 1993 and 1995, respectively. Similar to the clade I isolates, the clade II isolates were identified exclusively before or in 2005. Clade III was the most dominant clade and prevailed during 2000 and 2016. Among clade III, three sub-clades that, respectively, diverged in 1995, 2002, and 2003 were exclusively identified in northern Taiwan (marked with dark red in Figure 1), indicating local transmission or outbreaks of certain strains in addition to the nation-level epidemic of this ST239 clone.

#### ST59

**FIGURE 2:**
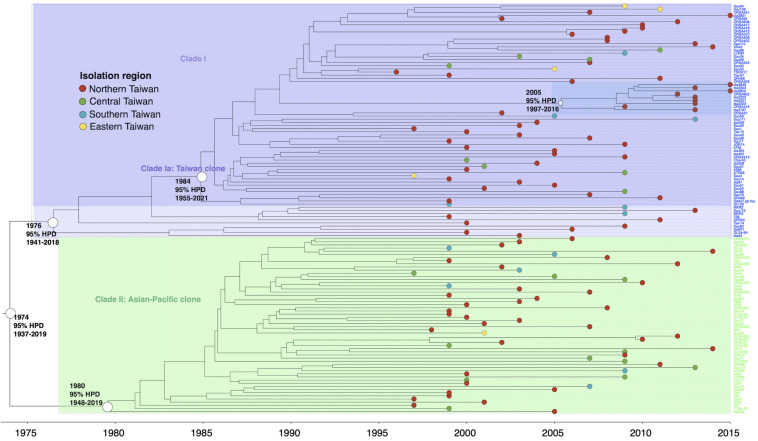
Time-scaled phylogeny of 129 methicillin-resistant *Staphylococcus aureus* isolates of the ST59 lineage collected in Taiwan during 1996 and 2016. Two major clades were marked with different colors. The hollow circles indicate the most recent common ancestors of isolates of the indicated clade and its estimated year of appearance. A subclade (marked as dark blue) in clade Ia that diverged in 2005 was exclusively identified in northern Taiwan, suggesting a local transmission of the strain. HPD, highest posterior density; SLV, single-locus variants.

The isolates of ST59 or its SLVs shared an MRCA in 1974 (95% HPD 1937–2019) and could be grouped into two major clades, respectively, of clade I (73 isolates, 56.6%) and clade II (56 isolates, 43.4%, Figure 2). The isolates of clade I shared an MRCA in 1976 (95% HPD 1941–2018) and further diverged in 1984. The daughter lineage clade Ia that descended from the MRCA in 1984 was collectively named as “Taiwan clone (TW clone)” in our previous studies, which could be differentiated from the clade II isolates named as “Asian-Pacific clone (AP clone)” not only phylogenetically but by the distinct genomic profiles in terms of the different distribution of genes of resistance and virulence (see below).

#### ST5

**FIGURE 3:**
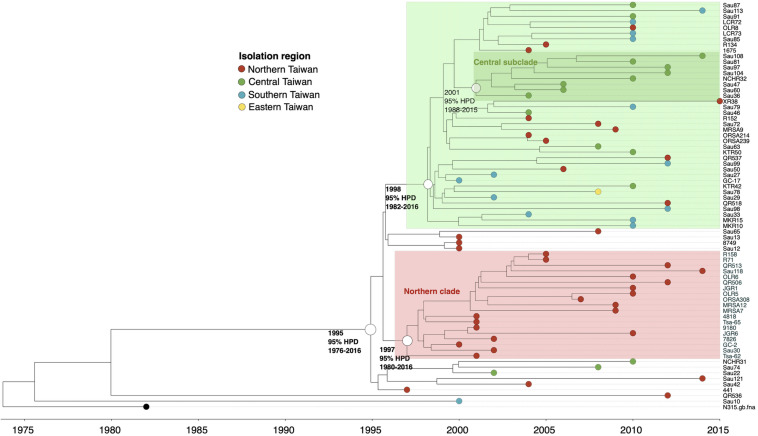
Time-scaled phylogeny of 71 methicillin-resistant *Staphylococcus aureus* isolates of the ST5 lineage collected in Taiwan during 1996 and 2016. The hollow circles indicate the most recent common ancestors of isolates of the indicated clade and its estimated year of appearance. HPD, highest posterior density; SLV, single-locus variants.

Except for two strains (QR536 and Sau10), a majority (97.2%) of the ST5 isolates were descendants of an MRCA appearing in 1995 (95% HPD 1976–2016, Figure 3). The ST5 isolates can be categorized into two major clades. Of them, a clade with isolates that diverged in 1997 was exclusively identified in northern Taiwan (northern clade, marked as red in Figure 3). The isolates of another clade were widely distributed in this island, though a subclade consisting of eight isolates that diverged in 2001 (marked as dark green in Figure 3) was exclusively identified in central Taiwan.

### Distribution of Virulence and Resistant Genes and Plasmids

#### ST239/ST241

**FIGURE 4:**
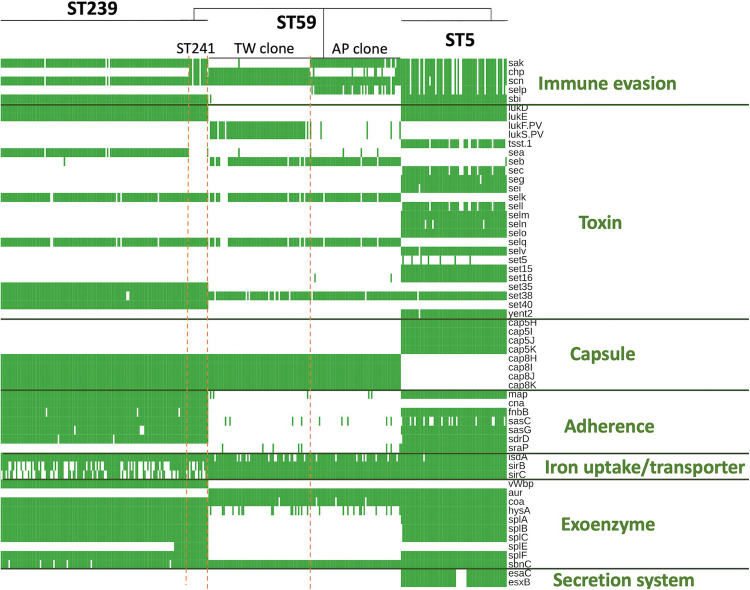
Differentially distributed virulence factors among methicillin-resistant *Staphylococcus aureus* isolates of three prevalent lineages, ST239/241, ST59, and ST5, in Taiwan.

**FIGURE 5:**
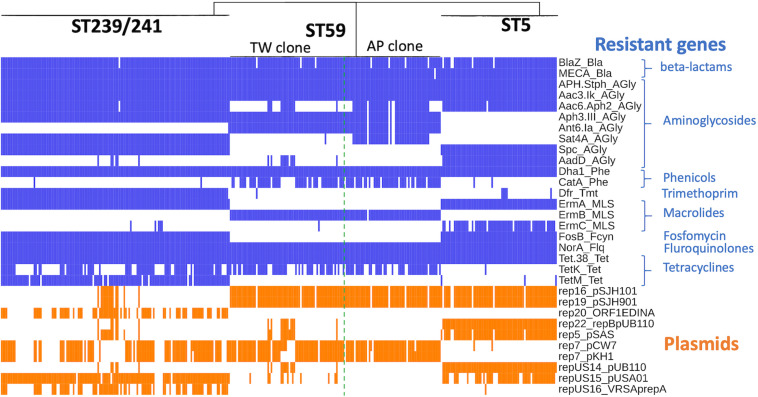
Distribution of genes mediating the antimicrobial resistance in methicillin-resistant *Staphylococcus aureus* isolates of three prevalent lineages, ST239/241, ST59, and ST5, in Taiwan.

The ST239/ST241 isolates carried a median (range) number of 118 (113–120) virulence genes and 16 (13–17) resistance genes (Supplementary File 1). Among them, three virulence genes including *chp*, *sea*, and *splE*, respectively, encoding chemotaxis inhibitory protein, staphylococcal enterotoxin A, and serin protease E were differentially distributed in the isolates of three different clades (Figure 4). The isolates of clade I (ST241 or its SLV) were mostly positive for *chp* (10/12, 83.3%) and were all negative for *sea*, whereas the isolates of clade II/III (ST239 or its SLVs) were all negative for *chp* but mostly positive for *sea* (100 and 97.4% for clade II and III isolates, respectively; Figure 4). Gene *splE* was carried by all isolates of clade I/II but missing in all isolates of clade III. The distribution of harbored resistant genes and plasmids was not significantly different for the isolates of the three clades (Figure 5).

#### ST59

**FIGURE 6:**
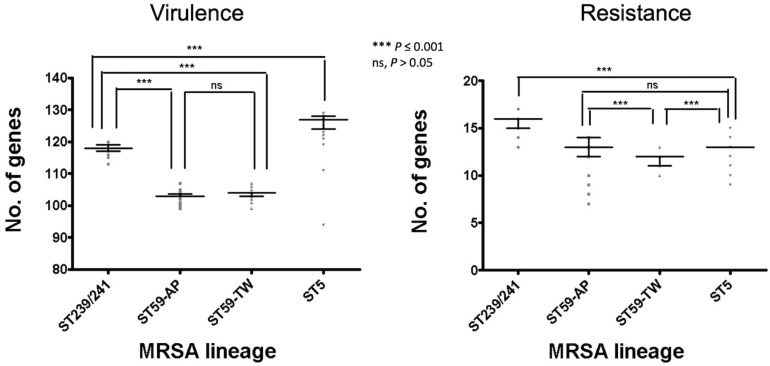
Comparison of the numbers of carried virulence and resistance genes among methicillin-resistant *Staphylococcus aureus* of different genetic lineages, including the Taiwan clone and the Asia-Pacific clone of ST59. The comparisons were conducted with one-way ANOVA method with Tukey’s multiple-comparison test.

Of the three lineages, the ST59 isolates harbored the smallest number of virulence and resistance genes (Figure 6). A median (range) of 103 (99–107) virulence genes and 12 (7–14) resistance genes was identified in ST59 isolates (Supplementary File 2). The number of carried virulence genes was similar in the two subclones of ST59 (*P* > 0.05, Figure 6). However, the TW clone carried a smaller number of resistance genes compared to the AP clone (mean ± standard deviation, 11.7 ± 0.73 *vs.* 12.8 ± 1.53, *P* < 0.001, Figure 6). The isolates of TW clone (clade Ia) were characterized with the carriage of a *ccrC2* gene (100%) belonging to SCC*mec* type V, *chp* (100%), and *lukF.PV* and *lukS.PV* encoding PVL (61/63, 96.8.0%) but mostly absent for *sak* encoding staphylokinase (1/63, 1.59%) and *selp* encoding enterotoxin P (0/73, Figure 4). The distribution of the toxemia profile listed above was complementary in the isolates of the AP clone (clade II), which carried *sak* (96.4%) and *selp* (78.6%) at high incidences but carried *chp* (12.5%) and PVL genes (5.36%) at relatively low rates. Furthermore, the isolates of AP clone did not harbor *ccrC2* (0/56). All of them harbored a type IV SCC*mec*.

Four replicons of plasmids were more commonly identified in the TW clone than in the AP clone, which included rep22_repBpUB110 (15.1% *vs.* 0, *P* = 0.0024), rep5_pSAS (17.8% *vs*. 0, *P* = 0.0005), repUS14_pUB110 (15.1% *vs*. 0, *P* = 0.0024), and repUS15_pUSA01 (15.1% *vs*. 0, *P* = 0.0024). The resistome disclosed that the alleles mediating the resistance to aminoglycosides, including Aac6_Aph2_AGly (76.8 *vs*. 17.8%, *P* < 0.0001) and Sat4A_AGly (76.8 *vs*. 1.37%, *P* < 0.0001), were more commonly identified in the AP clone, whereas AadD_AGly (15.1% *vs*. 0, *P* < 0.0001) was exclusively identified in the TW clone.

#### ST5

The ST5 isolates harbored the greatest number of virulence genes among the three lineages, with a median (range) of 127 (119–129) virulence genes (Figure 6 and Supplementary File 3). The toxemia profile carried by the plasmids and resistome was similar for the ST5 isolates irrespective of clade (Figures 4, 5). The virulence genes mediating immune evasion functions (*sak*, *chp*, *scn*, and *selp*), toxins (*tsst-1*, *sec*, *sell*, and *seln*), and adherence (*sasC*) were absent in some ST5 strains, but the absence was not confined to specific clades. Two virulence genes, *esaC* and *esxB*, accounting for the type VII secretion system were present in a majority of the ST5 strains but absent in the isolates of the central subclade.

### Lineage-Specific Distribution of Resistome, Plasmids, and Toxome

The resistome (Figure 5) of three lineages disclosed that the genes mediating the resistance to beta-lactams (*BlaZ* and *mecA*), aminoglycosides (*APH.Staph* and *Aac3.Ik*), phenicols (*Dha1*), floroquinolone (*NorA*), and tetracycline (*Tet.38*) were carried at extremely high rates (all >95%) by all isolates irrespective of genetic lineage. Gene *dfr*, mediating the resistance to trimethoprim, was identified in 99.3% of the isolates of ST239/241, whereas only 7% and 0.5% of the isolates of ST5 and ST59, respectively, harbored this gene. The *fosB* gene mediating the resistance to fosfomycin was identified in 100% of the isolates of ST 239/241 and ST5 but was completely absent in the isolates of ST59.

When detected with the replicon sequences, a total of 10 plasmids were identified in the MRSA isolates of three lineages. The isolates of different lineages harbored their own pattern of plasmids. Nonetheless, the isolates of a different clone can also carry similar plasmids. For instance, rep16_pSJH101 and rep19_pSJH901 were simultaneously carried by the isolates of ST5 and ST59 at incidences of 91.5 and 94.6%, respectively. It appeared that the kinds of plasmids were more consistently carried in the isolates of ST59 and ST5 than in the isolates of ST239/241 (Figure 5).

Figure 4 displays only the virulence genes which were differentially distributed among three different lineages. Approximately 80% (78.9–80.3%) of the ST5 isolates carried the four IEC genes, including *sak*, *chp*, *scn*, and *selp*. For the ST59 isolates, the carriage rates of *sak*, *chp*, and *selp* were relatively low (34.1–61.2%), but *scn* was carried by 99.2% of the isolates. A majority of the ST239/241 isolates carried *sak* and *scn* (96.4%) but absent for *chp* and *selp* (7.1% and 0, respectively). The *sbi* gene encoding immunoglobulin-binding protein was carried by all the isolates of ST5 and ST239/241 but absent in the isolates of ST59.

The toxin genes listed in Figure 4 were more frequently identified in the isolates of ST5 than in the isolates of the other two MRSA lineages—for instance, enterotoxin-like genes, including *sec*, *seg*, *sei*, *sell*, *selm*, *seln*, *selo*, *selv*, *sett15*, *set16*, and *yent2*, were carried at high rates in the ST5 isolates but completely absent in the ST59 and ST239/241 isolates. Gene *tsst-1*, encoding toxic shock toxin-1, was also exclusively identified in the ST5 isolates at a rate of 85.9%. *lukD/E*, encoding leukocidin D and E, was carried by all the isolates of ST5 and ST239/241 but not by any isolate of ST59. The PVL genes were exclusively identified in the isolates of ST59, especially the TW clone, with a rate of 96.8% (61/63).

The distribution of the capsule genes was identical for the ST239/241 and ST59 isolates. The genes mediating adherence and exoenzymes were more commonly identified in the isolates of ST5 and/or ST239/241 than in the isolates of ST59, which included *map*, *fnbB*, *isdA*, *sasC*, *sasG*, *sdrD*, *hysA*, *splA*, *splB*, *splC*, and *splF*. The genes *esaC* and *esxB*, involved in the type VII exertion system, were exclusively identified in the ST5 isolates.

## Discussion

The results from the current study demonstrated that, among the top three MRSA clones in Taiwan, ST59 was the earliest lineage appearing as early as in 1974 and rapidly diverged into two dominant clades in 1980 (AP clone) and 1984 (TW clone). The TW clone was prevailing in Taiwan and other regions during the global CA-MRSA pandemic in the late 1990s and 2000s (Huang and Chen, 2011; Chen et al., 2013; Feng et al., 2017). More than 90% of the CA-MRSA infections in Taiwan were caused by the TW clone during the epidemic (Huang et al., 2008; Chen and Huang, 2014). Similar to the microbiological features of most CA-MRSA in the world, the ST59 CA-MRSA was characterized with carriage of PVL genes and a smaller size of SCC*mec* element (V_*T*_). Information from the WGS analysis further revealed that the isolates of the ST59 TW clone were different from its sister clone, the AP clone, by carriage of *chp* gene and mostly absent for *sak* and *selp* of immune evasion function and two genes mediating aminoglycoside resistance (*Aac6.Aph2* and *Sat4A*).

It was intriguing to learn that the ancestor of ST59 strains might have been present in this island for more than a decade before being identified as the dominant clone of CA-MRSA. It was generally believed that acquiring certain virulence factors (i.e., PVL and phenol-soluble modulins) and/or increasing the expression of certain virulence factors (i.e., alpha-toxin) played a major role for the success of the CA-MRSA strains (Genestier et al., 2005; Wardenburg et al., 2007; Hongo et al., 2009; Peschel and Otto, 2013). The comparison of the toxome of the three MRSA lineages further indicated that the ST59 strains were distinct from the ST239/241 and ST5 strains by carrying less virulence genes. Indeed, a range of genes accounting for adherence (*map*, *fnbB*, *sasC*, *sasG*, *sdrD*, and *sraP*) and exoenzymes (*hysA*, *splA*, *splB*, *splC*, and *splF*) were absent in the ST59 strains. The *seb* gene mediating staphylococcal enterotoxin B was the only exception which was carried by ST59 but missing in the strains of the other two lineages.

Another well-known characteristic of global CA-MRSA was their limited resistance to non-beta-lactams. In line with this notion, our data indicated that the ST59 strains of TW clone carried the smallest number of resistance genes among the several epidemic clones (Figure 6). Furthermore, the ST59 strains, irrespective of the TW or AP clone, did not harbor resistant genes, including *dfr* mediating the resistance to trimethoprim and *fosB* mediating the resistance to fosfomycin. However, the ST59 strains were mostly positive for genes mediating the resistance to aminoglycoside, macrolides, phenicols, and tetracyclines. The ST59 CA-MRSA in Taiwan was phenotypically resistant to clindamycin and macrolides, which was different from the antibiograms in the other pandemic CA-MRSA lineages, including USA 300.

The AP clone of the ST59 MRSA that emerged in 1980 was characterized with the carriage of a type IV SCC*mec* and negative for PVL genes. The isolates of the AP clone were usually positive for the *sak* and *selp* genes. In our previous molecular epidemiology studies of CA-MRSA, the AP clone was identified as the most dominant MRSA clone colonizing healthy individuals (Huang et al., 2005; Chen et al., 2007). Recent data further demonstrated the isolation of MRSA of the CA genotype from hospitalized patients or patients with HA risk factors, suggesting the entry of the CA-MRSA strains into healthcare facilities and causing nosocomial infections (Huang et al., 2007). However, our data disclosed that the strains with CA genotype (i.e., carriage of SCC*mec*IV) might have been present as early as in 1980 in the hospitals in Taiwan, which was approximately 20 years before ST59 was recognized as a major cause of CA-MRSA infections in previously healthy individuals. The finding was supported by a molecular epidemiology study of early MRSA strains in northern Taiwan, which demonstrated that a substantial proportion of the nosocomial MRSA strains in the early years carried the type IV SCC*mec* element and with the pulsotypes consistent with the ST59 strains (Chen et al., 1999). Combined together, the observation strongly suggested that the ST59 strains were among the earliest MRSA strains in Taiwan. The evolution of ST59 was through two major pathways, and the strains were successfully adaptive to both the community and healthcare environments in this island.

The Hungarian clone (ST239/241) in Taiwan was estimated to emerge in 1979, which was approximately 5 years later than the emergence of the ST59 isolates. The finding was somewhat unexpected because ST239/241 was identified as the most predominant HA-MRSA clone in the early (1990s) MRSA strains in Taiwan by our group and others (Huang et al., 2004; Chen and Huang, 2005; Wang et al., 2007). The Hungarian clone (ST239/241) was first reported in MRSA isolates collected from Hungarian hospitals and accounted for 67% of the MRSA isolates during 1993 and 1998 (de Lencastre et al., 1997; de Sousa and Lencastre, 2004). ST239/ST241 became a very successful MRSA clone in Taiwan, which was responsible for the increasing incidences of nosocomial MRSA infections during the 1990s and predominated the HA-MRSA strains in the following decades. The finding from the current study further indicated that the isolates of this clone were of three clades belonging to ST241 (clade I) and ST239 (clades II and III). The ST241 isolates appeared to emerge as early as in 1989 but were of minority and finally disappeared during the Hungarian clone evolution. None of the ST241 isolates was identified after 2006 in this study. The ST241 isolates can be differentiated from ST239 by the absence of the *sea* gene mediating staphylococcal enterotoxin A and the carriage of the *chp* gene and the *splE* gene encoding serine protease. The clade III of ST239 appeared to be a more successful clone, as it comprised the majority of the isolates of the Hungarian clone. The clade III isolates appeared to emerge later than the clade I strains (in 1995) and dominated the HA-MRSA strains in the late 1990s and early 2000s. In a national survey in 2010, the ST239 isolates remained the most dominant clone among the MRSA blood isolates in Taiwan (Chen et al., 2014). The ST239 isolates were very prevalent, and at least three clusters of ST239 infections occurring in northern Taiwan were identified in the current study.

The ST5 clone was considered one of the two major ancestral MRSA lineages in the world (Oliveira et al., 2001). However, the results from the current study indicated that the entry of ST5 MRSA into Taiwan had not occurred until 1995. Consistent with this finding, a longitudinal study characterizing the MRSA blood isolates that were collected during 1995 and 2006 in northern Taiwan demonstrated the emergence of the ST5 strains in 1998 (Chen et al., 2009). Actually, the ST5 isolates remained rarely identified in the late 1990s but were increasingly identified during 2000 and 2010. Two national surveys of MRSA blood isolates, respectively, in 2000–2001 and 2010 disclosed that the incidences of the ST5-SCC*mec*II isolates increased from nearly zero to approximately 20% in 10 years (Huang et al., 2004; Chen et al., 2013). The predominance of the ST5 strains in Taiwan in the late 2010s was also demonstrated in another surveillance study, which disclosed a high rate (42%) of the ST5 strains among 38 nasal MRSA isolates from patients in intensive care units in 2008 (Chen et al., 2010). The rapid increase of the ST5 clone in Taiwan suggested a selective advantage of the strains in the hospital environments of this island since its emergence in 1995. Although WGS-based phylogeny divided the clone into several subclades (clusters) with isolates exclusively identified in certain regions of this island, there was no significant alteration of the genomic contents in terms of the distribution of virulence and resistant genes and plasmids among the available ST5 isolates during the two decades of evolution (1995–2015). The ST5 isolates carried more toxin genes (i.e., *sec*, *seg*, *sei*, *sell*, *selm*, *seln*, *selo*, *selv*, *set15*, *set16*, and *yent2*) when compared to the isolates of ST59 or ST239/241. The ST5 isolates were also positive for *sraP* mediating a cell-wall-anchored protein of adherence function and *esaC* and *esxB* genes accounting for type VII secretion system, which were absent in the isolates of the other two lineages.

## Conclusion

**FIGURE 7:**
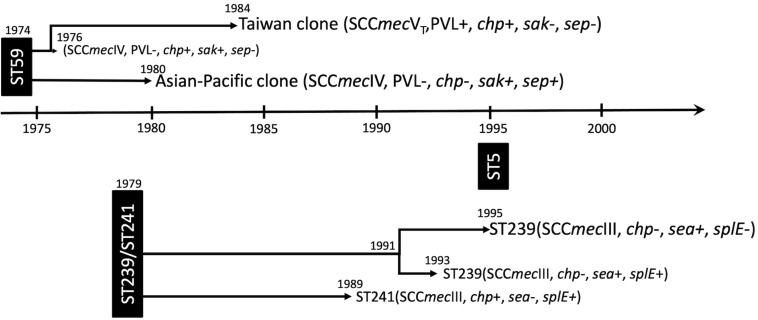
Summary of the evolution timeline and characteristics of isolates of three prevalent methicillin-resistant *Staphylococcus aureus* lineages in Taiwan.

Because of the unavailability of the actual locations for most of the MRSA strains, the study was limited by not having been able to perform a spatiotemporal analysis, and the dispersal dynamics between different regions and/or between hospitals and communities cannot be readily captured. The strength of this WGS study was able to comprehensively delineate the lineage-specific toxome, resistome, and plasmid profiles in the three prevalent MRSA lineages in Taiwan. The genomic contents were mostly conserved across distinct clones within the same lineage. Nevertheless, differentially distributed genes could be identified among those of immune evasion function, leukocidins, enterotoxins, and exoenzymes and genes mediating the resistance to aminoglycosides. The estimated year of emergence, evolution pathways, and timeline of genetic divergence of the three MRSA lineages were also comprehensively captured in this study and summarized in Figure 7. Of them, the ST59 lineage, traditionally known as the epidemic CA-MRSA clone in Asian countries, was estimated to emerge as early as in 1974 in Taiwan and diverged into two clones, namely, AP clone and TW clone, in the following 10 years. The strains of the AP clone with SCC*mec* IV negative for PVL genes soon became a significant cause of HA-MRSA infections in the 1980s and the most common CA-MRSA colonizing isolates since the late 1990s. The TW clone with SCC*mec*V_*T*_ positive for PVL genes accounted for more than 90% of CA-MRSA infecting isolates in Taiwan during the global CA-MRSA pandemic commencing in the late 1990s. Another early MRSA lineage was ST239/ST241 that emerged in 1979 and diverged into three different clones during 1989 and 1995. All of the three clones were once constituting the most prevalent HA-MRSA strains before 2010, but only the ST239-SCC*mec*III clone positive for *sea* and negative for *chp* and *splE* appearing in 1995 remained prevalent in the healthcare facilities in this island after 2010. The ST5 lineage emerged much later than ST59 and ST239/241in Taiwan, and no significant genomic evolution was identified after its emergence in 1995.

## Data Availability Statement

The datasets presented in this study can be found in online repositories. The names of the repository/repositories and accession number(s) can be found below: https://www.ncbi.nlm.nih.gov/, PRJNA695114.

## Author Contributions

C-JC and Y-CH designed the study. Y-CH and T-LY performed the samples and data collection. C-JC conducted the genome sequencing, data analysis, and writing of the manuscript. All authors contributed to the article and approved the submitted version.

## Conflict of Interest

The authors declare that the research was conducted in the absence of any commercial or financial relationships that could be construed as a potential conflict of interest.

## Publisher’s Note

All claims expressed in this article are solely those of the authors and do not necessarily represent those of their affiliated organizations, or those of the publisher, the editors and the reviewers. Any product that may be evaluated in this article, or claim that may be made by its manufacturer, is not guaranteed or endorsed by the publisher.

## Footnotes

1
https://github.com/rambaut/figtree/releases
